# Neurofibrillary tangle distribution in posterior cortical atrophy and typical early-onset Alzheimer's Disease

**DOI:** 10.17879/freeneuropathology-2026-9686

**Published:** 2026-07-09

**Authors:** Denis S. Smirnov, Sophie M. Dickinson, Melanie F. Estrella, Vanessa S. Goodwill, Alison J. Headley, Donald P. Pizzo, Doug Galasko, David P. Salmon, Annie Hiniker

**Affiliations:** 1 Department of Pathology, Mass General Brigham, Harvard Medical School, Boston, Massachusetts, USA; 2 Department of Neurosciences, School of Medicine, University of California, San Diego, La Jolla, California, USA; 3 Department of Pathology, School of Medicine, University of California, San Diego, La Jolla, California, USA; 4 Department of Pathology, Keck School of Medicine, University of Southern California, Los Angeles, California, USA; 5 Department of Pathology, Los Angeles General Medical Center, Los Angeles, California, USA

**Keywords:** Alzheimer's Disease, Posterior cortical atrophy, Tau, Neurofibrillary tangles, Cognitive impairment

## Abstract

**Background and objectives**: Posterior Cortical Atrophy (PCA) is a focal variant of Alzheimer's Disease (AD) characterized by disproportionate and early visuospatial deficits with relative sparing of other cognitive abilities. PCA commonly arises before age 65, consistent with early onset AD (EOAD). Because studies of PCA pathology in patients with detailed cognitive assessment are rare, relationships between severity and distribution of neurofibrillary tangle (NFT) density and visuospatial deficits in PCA are not fully known. Our objectives were to: (1) determine if ratio of NFT density in occipital cortex (OC) versus hippocampus (HC) is higher in PCA than typical EOAD and (2) determine if this ratio correlates with severity of visuospatial deficits in EOAD generally.

**Methods**: We determined density of tau NFT pathology in primary visual cortex (OC), midfrontal cortex (MF), and HC and calculated ratios of these densities for EOAD patients with PCA (n = 12) or non-PCA (n = 33) clinical phenotypes. Groups were compared using linear regression that adjusted for age at death and sex. Correlation between NFT density and visuospatial and other cognitive domain scores were examined using linear regression that adjusted for age at cognitive testing, education, sex and test-death interval. Analyses were repeated with the non-PCA EOAD patients grouped into those with mild (EOAD-Typical, n = 21) or severe (EOAD-Visual, n = 12) visuospatial impairment.

**Results**: PCA patients had lower HC (β = –11.7 ± 5.7, p = 0.045) and a trend toward higher OC (β = 6.9 ± 3.7, p = 0.07) NFT density, and higher OC/HC (β = 0.30 ± 0.14, p = 0.04) and OC/MF (β = 0.56 ± 0.16, p = 0.001) ratios, than typical non-PCA EOAD patients. The EOAD-Visual had higher OC/HC NFT ratios (β = 0.38 ± 0.13, p = 0.008) than the EOAD-Typical non-PCA patients even though they did not meet clinical criteria for PCA. Correlations between OC/HC NFT ratios and visuospatial domain scores were strong (β = –1.29 ± 0.45, p = 0.008) and remained significant when limited to the non-PCA EOAD group (β = –1.22 ± 0.43, p = 0.008).

**Discussion**: PCA is associated with a distribution of NFT pathology (i.e., high OC/HC NFT ratio) that coincides with early predominant visuospatial impairment. Non-PCA EOAD with a memory-predominant presentation and concomitant visuospatial impairment has an NFT distribution profile similar to PCA. NFT pathology in occipital cortex may moderate degree of visuospatial impairment in EOAD regardless of clinical syndrome.

## Introduction

 Alzheimer's disease (AD) typically begins with accumulation of pathology in hippocampus and entorhinal cortex^1,2^ leading to initial symptoms of memory impairment^3^. There is considerable heterogeneity in the clinical and neuropathologic presentation of AD, particularly in those with early-onset AD (EOAD, i.e., age of onset < 65 years). A significant minority of EOAD cases start with prominent neocortical involvement that produces relatively circumscribed deficits in various non-memory cognitive domains^4^. These atypical presentations are grouped clinically into syndromes describing specific core symptoms such as visuospatial deficits in Posterior Cortical Atrophy (PCA)^5,6^, language deficits in Primary Progressive Aphasia (PPA)^7,8^, or executive dysfunction in the frontal variant of AD^9,10^. These subtypes diverge early in the course of disease with disparate patterns of cortical tau deposition on tau-PET imaging corresponding to distinct cognitive phenotypes^11^. 

 PCA is a focal variant of AD characterized by relatively circumscribed atrophy of occipital cortex^5,12^. Symptoms begin insidiously with prominent and progressive visuospatial dysfunction that precedes or overshadows other cognitive deficits^6^. This visual-dominant presentation can have a non-AD etiology (e.g., Lewy body disease, corticobasal degeneration, Creutzfeldt-Jakob disease), but is most commonly caused by AD.^1,3^ Clinical PCA syndrome makes up ∼5–13 % of all EOAD cases^15,16^. In the largest cohort reported to date (n = 1092 across 36 centers), approximately 75 % of PCA patients had symptom onset before age 65 (mean 59.4 years), and PCA attributable to Alzheimer's disease skews younger still.^13^ The pronounced visuospatial deficits of PCA have been attributed to tau pathology in visual processing areas of occipital cortex; consistently, tau-PET imaging studies show atypically high AV1451 tau tracer signal across the occipital cortex of patients with PCA^17–19^. 

 The relationship between PCA clinical symptoms and regional distribution of tau neurofibrillary tangle (NFT) pathology has not been fully defined, particularly with respect to the relative involvement of occipital lobe compared to other regions. Early neuropathologic studies^20,21^ showed increased NFT pathology in occipital and inferior parietal cortex of PCA versus non-PCA patients, but both groups also had high tangle density in the hippocampus and paralimbic regions. Most of these PCA patients eventually developed both visuospatial and memory symptoms; however, relative degree of visuospatial and memory impairment was not clinically quantified. Tang-Wai et al.^22^ found that patients with PCA due to AD had significantly higher NFT density in occipital primary (Brodmann area [BA] 17) and secondary (BA 18) visual cortex than patients with typical AD, and significantly fewer NFTs in hippocampus and subiculum but examined general symptom profiles without quantitative assessment of defined cognitive domains. Other studies found an association between visuospatial abilities and NFT density in the angular gyrus but did not examine occipital cortex^23^. More recently, Abdi et al.^24^ used digital image analysis and found higher amyloid-beta (Aβ) and tau burden in the parietal cortex of PCA versus amnestic AD, without differences in occipital pathology. However, this study did not examine medial temporal lobe pathology, and did not relate the pathologic findings to clinical features. Thus, it remains unclear whether visuospatial impairment in PCA and related EOAD syndromes reflects absolute posterior cortical NFT burden, relative sparing of medial temporal structures, or the balance between regional pathologies. 

 Further complicating the picture, the relationship between visuospatial deficits and occipital AD pathology is not unique to the PCA phenotype but also occurs in typical memory-predominant presentations of AD that also have significant visuospatial impairment^25–28^. Ingram et al.^29^ plotted typical AD cases into the multidimensional space derived from principal components analyses of detailed neuropsychological test data from PCA cases (and vice versa). This analysis showed graded overlap along visuoperceptual, visuospatial and general cognitive impairment dimensions with little evidence to support stringent categorical clustering. Visuoperceptual and visuospatial deficits in both groups were associated with MRI-derived measures of grey matter volume loss in occipital cortical regions (e.g., lingual gyrus, intracalcarine cortex, lateral occipital cortex). Other studies have found faster metabolic decline in parietal and precuneus cortex in typical AD with prominent visuospatial impairment compared to those without^30^. These results suggest that AD and PCA may lie along a continuum of cognitive-neuroanatomical changes. 

 Here we expand on our prior work, in which we found that the relative distribution of NFT pathology across hippocampal and cortical regions (rather than absolute regional burden) tracked age-at-onset–related clinical heterogeneity in AD, with earlier-onset cases showing a more neocortical and less hippocampal-predominant tangle distribution.^31^ In the present study, we examine the relative density of NFT in occipital primary visual cortex versus hippocampus in patients with high AD neuropathological changes (ADNC) who had a clinical presentation of either PCA or typical EOAD. We test the hypotheses that patients with PCA have a higher occipital to hippocampal NFT density ratios than those with typical EOAD and that this ratio correlates with the severity of their visuospatial deficit. 

## Methods

### Participants

Participants were drawn from the UCSD Shiley-Marcos Alzheimer's Disease Research Center longitudinal clinical study and brain bank. Inclusion criteria included complete clinical evaluation within 5 years of death, high ADNC (NIA-AA criteria: Braak V–VI, Thal 4–5, CERAD moderate-frequent), and banked formalin-fixed tissue of hippocampus (HC), midfrontal (MF) cortex, and occipital (OC) cortex. Participants were excluded if they carried a concurrent pathologic diagnosis of Lewy body disease (limbic or neocortical) or frontotemporal lobar degeneration (FTLD) with tau, TDP-43, or FUS. Because PCA presents predominantly before age 65, we restricted both the PCA and AD comparison groups to early-onset cases (symptom onset < 65 years). This ensured the groups were comparable in age and minimized confounding by age-associated factors that could otherwise differ systematically between an early-onset-predominant syndrome and a typical late-onset AD population. To restrict the analysis to sporadic cases, we excluded those with a known pathogenic autosomal dominant mutation (e.g., PSEN1, APP) and those with a family history consistent with autosomal dominant inheritance.

### Clinical and neuropsychological evaluation

 PCA and non-PCA participants had received annual standardized clinical, neurological, and neuropsychological evaluations as previously described^31^. Clinical evaluation included review of history with the patient and/or informant, mental status testing, and assessment of functional impairment. Clinical Dementia Rating (CDR) score and sum of its six subdomain scores (i.e., CDR sum of boxes) were computed. Global cognition was measured by the Mini-Mental State Exam (MMSE) and the Dementia Rating Scale (DRS). Neuropsychological assessment included tests of *Memory* (Visual Reproduction Test, Logical Memory Test, California Verbal Learning Test (CVLT), CERAD Word List); *Language* (30-item Boston Naming Test, Letter Fluency Test (F-A-S), Category Fluency Test); *Executive functions* (modified Wisconsin Card Sorting Test, Trail Making Test Parts A and B, Digit Span Test); and *Visuospatial abilities* (Block Design Test, Visual Reproduction Test copy, Clock Drawing Test). A subset of these tests was used to generate Visuospatial, Memory, and Executive Function domain scores that were standardized based on the mean and standard deviation of domain scores from a robust normal control group of individuals who were cognitively normal at all UCSD Shiley-Marcos Alzheimer’s Disease Research Center (ADRC) evaluations (i.e., converted to z-scores), as previously described^31^. 

 Consensus clinical diagnoses were made according to published criteria by two board-certified neurologists who were blind to individual cognitive test scores but told whether neuropsychological assessment identified deficits in two or more cognitive domains. Probable AD, Possible AD, or Mild Cognitive Impairment (MCI) was diagnosed using NINCDS-ADRDA or NIA-AA criteria^32–34^. PCA was diagnosed according to consensus criteria^6^. Briefly, classification required: (1) insidious onset and gradual progression, (2) prominent early visual and/or visuospatial impairment in the absence of primary ocular disease, (3) relative sparing of anterograde memory, speech/language, behavior, and personality early in the course, and (4) absence of an alternative cause (e.g., tumor, stroke). 

### Neuropathological evaluation

 Autopsy was performed as previously described^35^. Brains were divided sagittally and the left hemibrain was fixed in 10 % buffered formalin, cut serially into 1 cm slices, and H&E-stained diagnostic sections taken, including MF, OC, HC, superior temporal cortex, inferior parietal cortex, entorhinal cortex, basal ganglia, midbrain with substantia nigra, pons with locus coeruleus, and cerebellum. 

***AD Pathology. ***Neuritic plaques, diffuse plaques, and NFTs were identified with immunohistochemical (IHC) staining for Aβ (Ab69D, rabbit polyclonal from Edward Koo, 1:1200) and paired helical filament (PHF) tau (PHF1, from Peter Davies, 1:600) on 5 μm-thick sections. Regional spread of amyloid pathology defined Thal phase^36^. Neuritic plaque density was estimated using Consortium to Establish a Registry for AD (CERAD) protocol^37^. Tau NFT pathology was staged according to the scheme of Braak and Braak^1^. Pathological diagnosis of AD was made using NIA-AA consensus criteria for the postmortem diagnosis of AD, wherein Thal phase 4–5, Braak stage V–VI, and moderate to severe neuritic plaque density corresponds to "high likelihood" of AD^38^. 

 HC, MF, and OC NFT densities were measured as previously described^31^. Blinded tau NFTs per high-power microscopic field ("NFT burden") were counted by two independent observers for each case in the areas of heaviest pathologic burden in MF, CA1 sector of HC, and primary visual OC. Counts were performed in three high-power fields per region and averaged to provide a single NFT count per 0.1 mm^2^ microscopic field. Ratios of regional counts (which provide information about the *relative* rather than absolute pathologic distribution) were calculated from raw counts, resulting in continuous unitless variables. 

***Non-AD pathology***. Lewy body pathology identified by H&E staining and α-synuclein IHC (phospho-synuclein 81A, from Virginia Lee, 1:15,000) and was staged as "brainstem", "limbic" (transitional), or "diffuse (neocortical)" according to consensus guidelines^39^, with limbic and neocortical LBD serving as an exclusion criterion. TDP-43 pathology was evaluated by immunohistochemical staining (Proteintech#10782-2-AP polyclonal, 1:12,000) and staged as "amygdala", "hippocampal", or "neocortical" according to Limbic-predominant Age-related TDP-43 Encephalopathy (LATE) guidelines^40^. Hippocampal sclerosis (HS) was diagnosed independent of TDP-43 pathology when neuronal loss in CA1 and subiculum was out of proportion with the degree of AD pathology. Vascular pathology was assessed by examining the brain for large arterial and lacunar infarcts, microinfarcts, and hemorrhages. Arteriolosclerosis, atherosclerosis of the circle of Willis, and cerebral amyloid angiopathy were each rated as "none", "mild", "moderate", or "severe" using a semi-quantitative scale. Atherosclerosis of the circle of Willis was graded grossly based on degree of luminal stenosis and vessel-wall change, while arteriolosclerosis was graded microscopically based on degree of arteriolar wall hyalinization/thickening and luminal narrowing in deep white matter and basal ganglia. Cerebral amyloid angiopathy was graded based on the extent and regional distribution of amyloid deposition within leptomeningeal and/or parenchymal vessels. 

### Statistical procedures

Demographic and clinical information for PCA and non-PCA EOAD patients was compared using Welch (unequal variance) t-tests and Fisher's exact tests for continuous and ordinal variables, respectively. Regional NFT counts were compared among groups using linear regression analyses adjusted for the participant's age at death and sex. When three groups were compared, post-hoc pair-wise group comparisons were made with Tukey's HSD tests. Correlations between regional NFT density measures and cognitive variables were evaluated using linear regression adjusted for sex, years of formal education, age at time of evaluation, and interval from evaluation to death. To confirm that observations were not driven by outliers in the PCA group, these regression analyses were repeated exclusively in the non-PCA EOAD participants. These models were further repeated with APOE ε4 carrier status (≥ 1 ε4 allele) and TDP-43 status (any stage) added as covariates. Statistical significance threshold (alpha) was set at *p *< 0.05. Analyses were performed using Statistical Package for Social Sciences (SPSS) Version 28 and R for Windows Version 4.3.2.

### Standard protocol approvals, registrations, and patient consent

The research protocol was reviewed and approved by the UCSD Human Subjects Review Board. Informed consent was obtained from all patients or their caregivers consistent with California State law.

### Data availability

De-identified data will be made available by request from any qualified investigator.

## Results

### Cohort characteristics

 We identified 12 patients with sporadic EOAD (age < 65 at onset) meeting clinical criteria for PCA^6^ with high ADNC. A comparison group of 33 sporadic EOAD patients with ADNC not meeting clinical criteria for PCA was identified (non-PCA). PCA and non-PCA groups were well-matched on reported age of disease onset (p = 0.99; overall mean ± SD = 57.6 ± 3.7 years), age at baseline clinical evaluation (p = 0.78; 63.3 ± 4.7 years), and age at death (p = 0.92, 69.3 ± 5.6 years) (**Table 1**). Consequently, time interval between disease onset and death (i.e., duration, p = 0.89, overall mean ± SD = 11.7 ± 3.9 years) or between baseline clinical evaluation and death (p = 0.78, 5.9 ± 2.6 years) was not significant. The PCA and non-PCA groups did not significantly differ in sex distribution (p = 0.10; overall 38 % female). As has been reported, PCA patients had, on average, 3.7 more years of formal education (17.7 ± 1.4 years) than non-PCA patients (14.0 ± 2.6 years, p = 5.9x10^–7^). PCA patients were less likely to harbor the APOE ε4 risk allele (8 %) than non-PCA patients (67 %; p = 0.002), consistent with other studies of PCA^34^ and with the observation that atypical (non-memory) presentations of dementia largely occur in APOE ε4 negative patients^4^. 

### Clinical presentation of patients with PCA and Non-PCA EOAD

Initial clinical complaints involved cognition in 100 % of non-PCA patients and 83 % of PCA patients (p = 0.07), with two (17 %) of PCA patients presenting with complex motor symptoms (one with unexplained left arm myoclonus rapidly followed by visuospatial deficits; the other with difficulty using a keyboard). All PCA patients reported visuospatial changes as their first cognitive symptom (by definition), whereas 88 % of the non-PCA patients first reported memory deficits; 6 %, language concerns; and 3 %, executive dysfunction. One non-PCA patient (3 %) presented first with visuospatial symptoms but did not meet full clinical criteria for PCA due to concomitant significant deficits in short-term memory. None of the non-PCA patients met formal criteria for another focal cortical syndrome (i.e., primary progressive aphasia or behavioral/dysexecutive variant AD).

 All PCA and non-PCA patients were diagnosed with dementia at their baseline ADRC evaluation an average of 5.9 ± 2.6 years before death. The groups did not differ significantly on CDR-sum of boxes (p = 0.68) scores at their baseline evaluation (see **Table 1**). PCA patients scored an average of 4.9 points better than non-PCA patients on the MMSE (p = 0.007) and 12.2 points better on the DRS (p = 0.03), although it should be noted that these tests are heavily weighted towards orientation and memory while providing very little assessment of visuospatial ability. Despite similar levels of global impairment and time since estimated onset of symptoms (**Table 1**), PCA patients scored significantly worse than non-PCA patients on most baseline neuropsychological tests of visuospatial function, but better on tests of memory, language and executive function (**Table 2**). Neuropsychological test scores were used to generate Visuospatial, Memory, and Executive Function domain scores that were standardized based on the mean and standard deviation of domain scores from a robust normal control group (i.e., converted to z-scores), as previously described^33^. The loadings of individual tests in each domain are presented in **Table 2**. 

**Table 1:** Participant demographics, clinical characteristics and neuropathology

	**Early onset AD (N = 33)**	**PCA (N = 12)**	**P value**
Age at evaluation	63.46 ± 4.40	62.95 ± 5.69	0.78
Age at onset	57.61 ± 3.28	57.58 ± 4.87	0.99
Age at death	69.32 ± 5.41	69.11 ± 6.41	0.92
Baseline – death interval	5.86 ± 2.43	6.15 ± 3.16	0.78
Duration (onset to death)	11.72 ± 3.92	11.52 ± 3.9	0.89
Female	15 (45 %)	2 (17 %)	0.10
Education	14.03 ± 2.63	17.67 ± 1.37	**5.9 x 10 – 7**
APOE ε4 alleles: 0 /1 / 2	11 / 19 / 3 (33 % / 58 % / 9 %)	11/ 1 / 0 (92 % / 8 % / 0 %)	**0.002**
First type of symptom at presentation: cognition / behavioral / motor	33 / 0 / 0 (100 % / 0 % / 0 %)	10 / 0 / 2 (83 % / 0 % / 17 %)	0.07
Predominant cognitive deficit at presentation: memory / language / executive / visuospatial	29 / 2 / 1 / 1 (88 % / 6 % / 3 % / 3 %)	0 / 0/ 0 / 12 (0 % / 0 % / 0 % / 100 %)	**4.5 x 10 – 10**
Clinical diagnosis: AD / LBD / other	29 / 4 / 0 (88 % / 12 % / 0 %)	9 / 1 / 2 (75 % / 8 % / 17 %)	0.08
MMSE	20.09 ± 5.53	25.00 ± 4.43	**0.007**
DRS	106.48 ± 17.59	118.73 ± 13.86	**0.03**
CDR sum of boxes	6.35 ± 3.13	5.56 ± 5.31	0.68
Severe AD (Thal 4–5, Braak V–VI, CERAD mod.-frequent)	33 (100 %)	12 (100 %)	(definitional)
Lewy Body Disease: none / brainstem / limbic / neocortical	30 / 3 / 0 / 0 (91 % / 9 % / 0 % / 0 %)	11 / 1 / 0 / 0 (92 % / 8 % / 0 % / 0 %)	0.99
TDP-43: none / amygdala / limbic / neocortical	23 / 6 / 4 / 0 (70 % / 18 % / 12 % / 0 %)	12 / 0 / 0 / 0 (100 % / 0 % / 0 % / 0 %)	0.14
Cerebral amyloid angiopathy: none / mild / moderate / severe	2 / 8 / 16 / 7 (6 % / 24 % / 48 % / 21 %)	2 / 1 / 7 / 2 (17 % / 8 % / 58 % / 17 %)	0.53
Infarct/microinfarct	4 (12 %)	2 (17 %)	0.65
Atherosclerosis of the circle of Willis: none / mild / moderate / severe	15 / 10 / 6 / 2 (45 % / 30 % / 18 % / 6 %)	5 / 6 / 0 / 1 (42 % / 50 % / 0 % / 8 %)	0.32
Arteriolosclerosis: none / mild / moderate / severe	22 / 6 / 4 / 1 (67 % / 18 % / 12 % / 3 %)	8 / 2 / 2 / 0 (67 % / 17 % / 17 % / 0 %)	0.99

Demographic characteristics (mean and standard deviation), apolipoprotein E (APOE) ε4 status (frequency of ε4 alleles), duration of disease (years) and interval between baseline assessment and death (years) (mean and standard deviation), first type of symptom and predominant cognitive deficit at presentation (frequency of each), clinical diagnosis (frequency of Alzheimer’s disease (AD), Lewy body dementia (LBD), or other) and scores (mean and standard deviation) on the Mini-Mental State Exam (MMSE), Dementia Rating Scale (DRS) and Clinical Dementia Rating (CDR) sum of boxes at baseline shown separately for participants with early onset (typical) AD or posterior cortical atrophy (PCA). Also shown are the number (and percentage) of individuals in each group with severe AD pathology, infarct / microinfarcts, or various levels of Lewy body disease, TDP-43, cerebral amyloid angiopathy, atherosclerosis, or arteriolosclerosis pathology at autopsy. P-values for group comparisons with unequal variance t-tests or Fisher exact tests are shown.

**Table 2:** Cognitive performance and domain weights

**Test**	**EOAD**	**PCA**	**p**	**Memory**	**Visuospatial**	**Executive**
Logical Memory Immediate	5.91 ± 4.45	17 ± 8.94	0.009	0.85	0.25	0.26
Logical Memory Delayed	1.75 ± 2.85	13.67 ± 8.09	0.002	0.89	0.16	0.19
Visual Reproduction Immediate	5.08 ± 2.8	3.44 ± 3.43	0.224	0.69	0.51	0.21
Visual Reproduction Delayed	1.08 ± 1.18	1.67 ± 2.55	0.525	0.82	0.32	0.11
CVLT Trials 1–5	18.28 ± 7.07	30.22 ± 7.64	0.001	0.85	0.30	0.26
CVLT Free Recall	2.28 ± 2.01	7.67 ± 4.61	0.008	0.90	0.20	0.16
CVLT Recognition	0.54 ± 0.12	0.73 ± 0.15	0.007	0.84	0.24	0.17
CERAD Word List Immediate	7.86 ± 4.08	14.78 ± 5.67	0.006	0.79	0.35	0.29
CERAD Word List Delayed	0.89 ± 1.29	3.33 ± 2.74	0.029	0.89	0.22	0.14
CERAD Word List Recognition	0.75 ± 0.14	0.91 ± 0.14	0.012	0.69	0.27	0.18
Visual Reproduction Copy	13.04 ± 3.52	6.78 ± 4.74	0.004	0.14	0.88	0.09
Block Design	13.39 ± 15.06	4.2 ± 4.29	0.004	0.45	0.73	0.27
Trail Making A	85.4 ± 48.75	135 ± 20.95	< 0.001	–0.30	–0.81	–0.22
Digit Span (Z-score)	–0.36 ± 0.7	0.23 ± 1.05	0.124	0.15	0.17	0.90
WCST Correct	23.39 ± 8.79	31.67 ± 8.67	0.029	0.42	0.50	0.42
Letter Fluency	23.79 ± 11.83	42.73 ± 16.57	0.004	0.41	0.38	0.59

Raw scores (mean ± standard deviation) on 16 neuropsychological tests administered at baseline shown separately for participants with early onset AD (EOAD) and posterior cortical atrophy (PCA). Tests include measures of memory (Logical Memory immediate and delayed recall; California Verbal Learning Test (CVLT) trials 1–5, free recall, and recognition; CERAD Word List immediate, delayed, and recognition; Visual Reproduction immediate and delayed recall), executive function (Wisconsin Card Sorting Test (WCST) correct responses, Letter Fluency, Digit Span), and visuospatial ability (Visual Reproduction copy condition, Block Design, Trail Making Test Part A). P-values for group comparisons with unequal variance t-tests are shown. Also shown are the loadings for each test on the three cognitive composite scores — Memory, Visuospatial, and Executive — derived from principal component analysis of the full set of 16 tests. Loadings reflect the weight of each test's contribution to the corresponding composite; tests with larger absolute loadings contribute more strongly to that composite.

### Distribution of NFT pathology in PCA and non-PCA EOAD

**Figure 1: Neurofibrillary Tangle Burden and Distribution in PCA vs typical EOAD:**
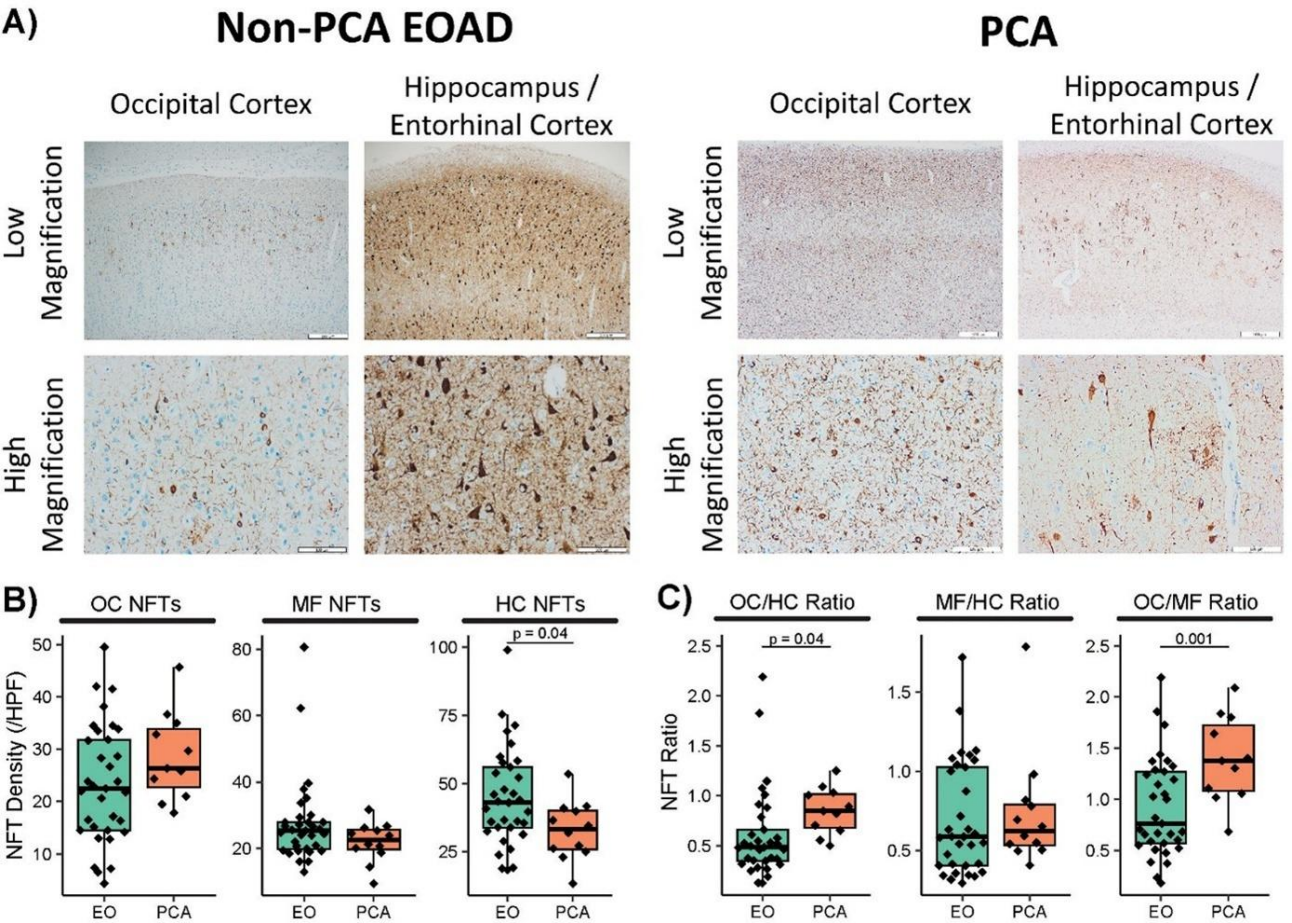
**A.** Example low and high magnification micrographs from representative fields of occipital cortex and hippocampus/entorhinal cortex for a participant with posterior cortical atrophy (PCA) (right panels) and a participant with non-PCA (i.e., typical) early onset Alzheimer's disease (left panels). **B.** Average neurofibrillary tangle (NFT) density per high power field (HPF) in the occipital cortex (OC), midfrontal cortex (MF), and hippocampus (HC) of those with early onset (EO) typical Alzheimer's disease or posterior cortical atrophy (PCA). **C.** Average ratio of neurofibrillary tangle density per high power field (NFT Ratio) comparing occipital cortex to hippocampus (OC/HC Ratio), midfrontal cortex to hippocampus (MF/HC Ratio), and occipital cortex to midfrontal cortex (OC/MF Ratio) of those with early onset (EO) typical Alzheimer's disease or posterior cortical atrophy (PCA). Scale bars (white) are 100 microns

 We first quantified NFTs in HC, MF, and OC in our cohort of patients with clinical diagnosis of PCA or non-PCA (typical) EOAD. **Figure 1A** shows example micrographs from representative fields of HC and OC of PCA versus non-PCA EOAD. Linear regression models that adjusted for age at death and sex showed that PCA patients had lower HC NFT density (β = –11.7 ± 5.7, p = 0.045) and trend-level higher OC NFT density (β = 6.9 ± 3.7, p = 0.07) compared to non-PCA EOAD patients (**Figure 1B**). The two groups did not differ in MF NFT density (p = 0.19). Adopting our previously applied methods^31^, we calculated ratios of NFT densities across pairs of anatomic regions (OC/HC, OC/MF, and MF/HC) to examine relative tau pathology distribution. Models that adjusted for age at death and sex showed that OC/HC (β = 0.30 ± 0.14, p = 0.04) and OC/MF (β = 0.56 ± 0.16, p = 0.001) NFT ratios were significantly greater in PCA than non-PCA EOAD patients, while MF/HC NFT ratios (p = 0.80) did not differ between these groups (**Figure 1C**). This pattern is consistent with the hypothesis that disproportionately high OC NFT pathology may give rise to the clinical profile of PCA (i.e., initial predominant visuospatial impairment). It should be noted, however, that a subset of the non-PCA EOAD patients also had high OC NFT density and high OC/HC and OC/MF NFT ratios but did not meet full clinical criteria for PCA; specifically, all except one presented with non-visuospatial deficits as first cognitive symptom and as such were not diagnosed as PCA. 

### Concomitant non-AD neuropathologies

DLB patients frequently show visuospatial abnormalities; therefore, Lewy pathology in neocortical or limbic regions, but not brainstem or amygdala, was an exclusion factor for this study. Brainstem Lewy pathology was present in ∼8–9 % of both PCA and non-PCA EOAD groups (p = 0.99). Groups did not differ with respect to cerebrovascular disease as measured by presence of at least one infarct or microinfarct (p = 0.65), atherosclerosis (p = 0.32), arteriolosclerosis (p = 0.99), or amyloid angiopathy (p = 0.53). There was no TDP-43 pathology in the PCA group, but 18 % of the non-PCA sporadic EOAD patients had amygdala-restricted TDP-43 pathology and 12 % had hippocampal TDP-43 pathology (p = 0.14). Across the cohort, TDP-43 pathology was associated with higher HC NFT density (p = 0.005) and MF density (p = 0.002), but not OC NFT density or any of the regional ratios.

### Baseline cognition and distribution of NFT pathology in PCA and non-PCA EOAD

**Figure 2:**
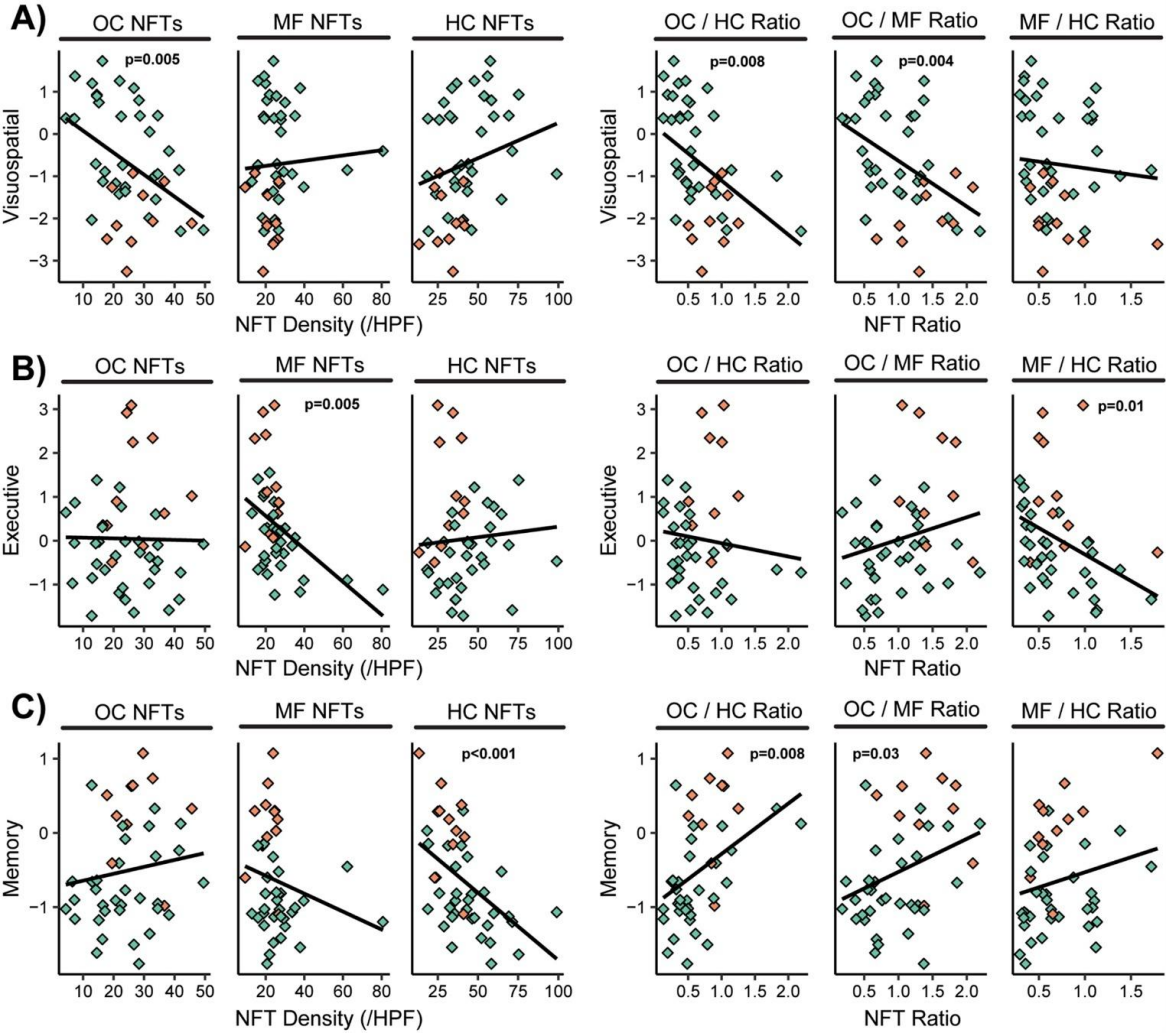
**Row A**: scatter plots of neurofibrillary tangle (NFT) density per high power field (HPF) in occipital cortex (OC), midfrontal cortex (MF), and hippocampus (HC), as well as NFT density ratios comparing OC to HC (OC/HC Ratio), MF to HC (MF/HC Ratio), and OC to MF (OC/MF Ratio), versus cognitive domain z-scores for visuospatial abilities. **Row B**. Same tangle densities and ratios versus executive function z-scores. **Row C**. Same tangle densities and ratios versus memory domain z-scores. Significant p-values are displayed from linear models adjusted for age at assessment, sex, education, and the interval between the baseline assessment and death (years). Full beta coefficients and p values for all comparisons are shown in **Table 3**, along with the same comparisons restricted to typical EOAD. Individuals with posterior cortical atrophy (PCA) are shown in orange and individuals with non-PCA (i.e., typical) early onset Alzheimer's disease are shown in green.

Linear regression analyses that adjusted for age at time of evaluation, education, sex, and interval between the evaluation and death were used to examine relationships between various cognitive domain scores and regional NFT density measures or NFT ratios. Across the combined cohort (PCA and non-PCA EOAD), lower (worse) Visuospatial domain scores were associated with higher OC NFT density (β = –0.052 ± 0.018, p = 0.005) and higher OC/HC (β = –1.29 ± 0.45, p = 0.008) and OC/MF (β = –1.08 ± 0.35, p = 0.004) NFT ratios (see **Figure 2, Table 3**). Lower Memory domain scores were associated with higher HC NFT density (β = –0.021 ± 0.006, p < 0.001) and lower OC/MF (β = 0.45 ± 0.20, p = 0.03) and OC/HC (β = 0.68 ± 0.24, p = 0.008) NFT ratios. Lower Executive Function domain scores were associated with higher MF NFT density only (β = –0.04 ± 0.01, p = 0.005) and higher MF/HC NFT ratios (β = –1.14 ± 0.43, p = 0.01).

**Table 3:** Association of regional tangle burden and cognitive performance

**Domain**	**Predictor**	**β ± SE (All)**	**p (All)**	**β ± SE (EOAD)**	**p (EOAD)**
Memory	**HC Tangles**	**–0.021 ± 0.006**	**< 0.001**	**–0.013 ± 0.006**	**0.031**
Memory	MF Tangles	–0.014 ± 0.009	0.126	–0.008 ± 0.009	0.378
Memory	OC Tangles	0.009 ± 0.01	0.375	0.01 ± 0.011	0.393
Memory	MF/HC Ratio	0.461 ± 0.293	0.124	0.255 ± 0.288	0.383
Memory	**OC/HC Ratio**	**0.674 ± 0.241**	**0.008**	**0.566 ± 0.223**	**0.017**
Memory	**OC/MF Ratio**	**0.449 ± 0.197**	**0.029**	0.372 ± 0.221	0.103
Executive	HC Tangles	0.004 ± 0.01	0.687	0.016 ± 0.008	0.055
Executive	**MF Tangles**	**–0.04 ± 0.013**	**0.005**	**–0.029 ± 0.01**	**0.01**
Executive	OC Tangles	–0.001 ± 0.017	0.942	–0.013 ± 0.015	0.376
Executive	**MF/HC Ratio**	**–1.137 ± 0.428**	**0.012**	**–1.141 ± 0.33**	**0.002**
Executive	OC/HC Ratio	–0.277 ± 0.433	0.527	–0.577 ± 0.319	0.082
Executive	OC/MF Ratio	0.493 ± 0.336	0.152	0.194 ± 0.314	0.542
Visuospatial	HC Tangles	0.017 ± 0.012	0.161	0.006 ± 0.012	0.625
Visuospatial	MF Tangles	0.006 ± 0.017	0.74	–0.004 ± 0.017	0.813
Visuospatial	**OC Tangles**	**–0.052 ± 0.018**	**0.005**	**–0.059 ± 0.019**	**0.004**
Visuospatial	MF/HC Ratio	–0.325 ± 0.536	0.548	–0.241 ± 0.573	0.677
Visuospatial	**OC/HC Ratio**	**–1.288 ± 0.454**	**0.008**	**–1.225 ± 0.429**	**0.008**
Visuospatial	**OC/MF Ratio**	**–1.079 ± 0.356**	**0.004**	**–1.123 ± 0.404**	**0.01**

Standardized regression coefficients (β) and p-values from linear regression models examining the association between neurofibrillary tangle (NFT) density (tangles per high-power field, /HPF) in the hippocampus (HC), middle frontal gyrus (MF), and occipital cortex (OC), and NFT density ratios (MF/HC, OC/HC, OC/MF), with composite scores for Memory, Executive, and Visuospatial cognitive domains. Cognitive composites were derived from principal component analysis of 16 neuropsychological tests administered at baseline (Table 2). All models were adjusted for age at assessment, sex, education, and the interval between the baseline assessment and death (years). Results are shown separately for the full cohort (early onset AD and posterior cortical atrophy combined) and for participants with early onset (typical) AD only.

These associations were not driven solely by the PCA patients. When these analyses were repeated with only non-PCA (typical EOAD) patients, results were remarkably similar. Visuospatial domain scores remained negatively associated with OC NFT density (p = 0.004) and OC/HC (p = 0.008) and OC/MF NFT ratios (p = 0.01). Similarly, Memory domain scores remained negatively associated with HC NFT density (p = 0.03) and OC/HC NFT ratio (p = 0.02), while Executive Function domain scores remained associated with MF NFT density (p = 0.01) and the MF/HC NFT ratio (p = 0.002) (see **Table 3**).

Because APOE ε4 and TDP-43 pathology are each associated with tau burden, we repeated the cognition–NFT analyses with APOE ε4 carrier status (≥1 ε4 allele) and TDP-43 status (any stage) added as covariates (**Supplemental Table 1**). All primary associations were retained: lower Visuospatial domain scores remained associated with higher OC NFT density (p = 0.007) and higher OC/HC (p = 0.02) and OC/MF (p = 0.02) NFT ratios, lower Memory scores were associated with higher HC NFT density (p = 0.01), and lower Executive scores were associated with higher MF NFT density (p = 0.02) and MF/HC ratio (p = 0.01). Results in the non-PCA EOAD group alone were comparable. Thus, the relationship between NFT burden and domain-specific cognitive impairment is not accounted for by APOE ε4 or TDP-43 status.

### Visuospatial impairment and OC tangle density in non-PCA EOAD

**Figure 3:**
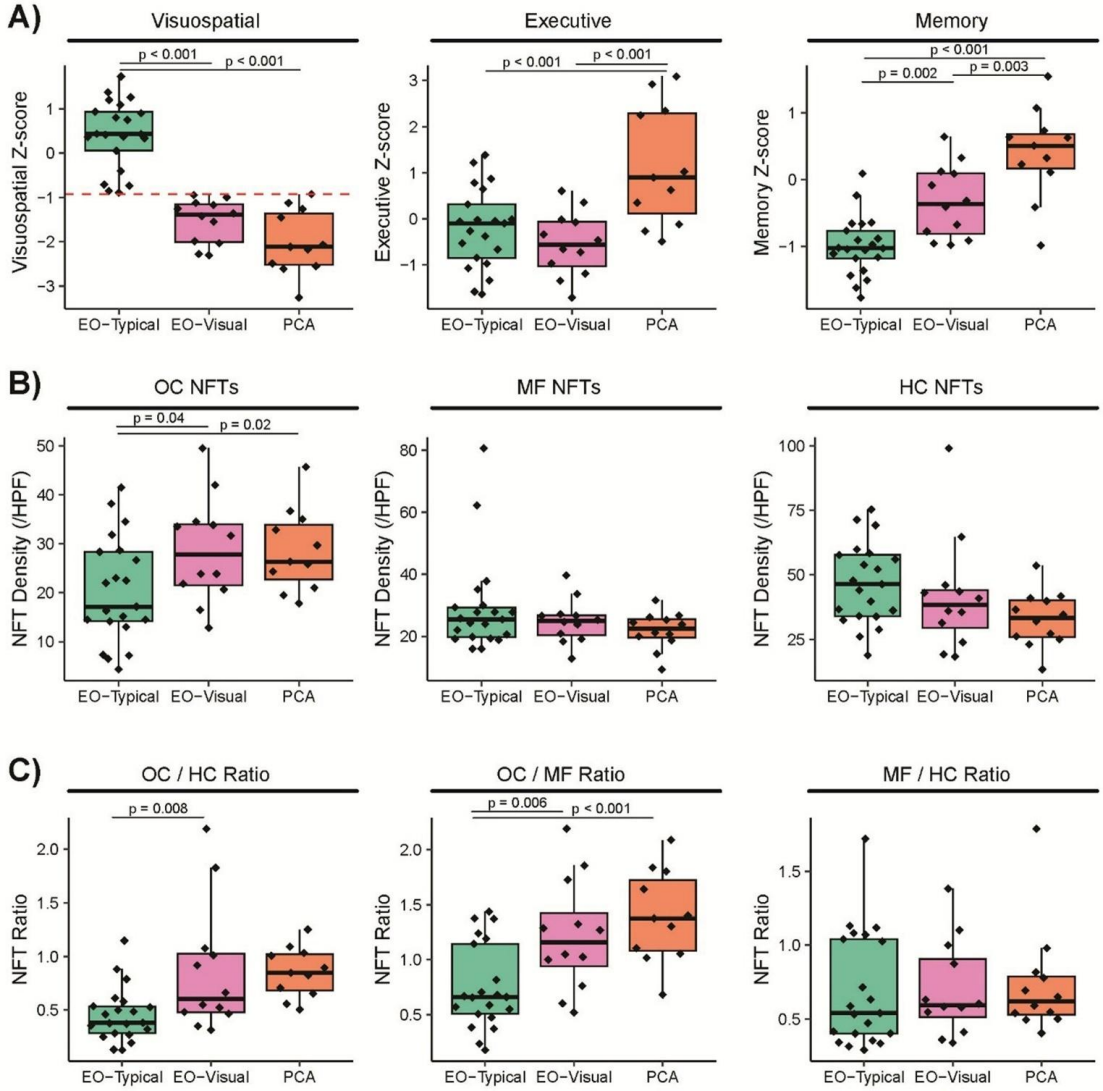
**Row A**: Distribution of visuospatial, executive, and memory cognitive domain z-scores for participants with posterior cortical atrophy (PCA) or non-PCA (i.e., typical) early onset Alzheimer's disease (AD) with (EOAD-Visual) or without (EOAD-Typical) significant visuospatial deficits (defined as visuospatial performance within the range of the PCA participants, red dashed line). **Row B**: Average neurofibrillary tangle (NFT) density per high power field (HPF) in the occipital cortex (OC NFTs), midfrontal cortex (MF NFTs), and hippocampus (HC NFTs) of those with PCA, EOAD-Visual AD or EOAD-Typical AD. **Row C**: Average NFT density ratios comparing OC to HC (OC/HC Ratio), OC to MF (OC/MF Ratio) and MF to HC (MF/HC Ratio) for those with PCA, EOAD-Visual AD or EOAD-Typical AD.

Given the negative association between Visuospatial domain scores and occipital NFT density, as well as with OC/HC and OC/MF ratios, in non-PCA EOAD patients, we divided the non-PCA EOAD patients into those with Visuospatial domain scores above ("EOAD-Typical") or in the same range ("EOAD-Visual") as our PCA patients to compare their clinical and neuropathologic features **(Figure 3A)**. The PCA (n = 12), EOAD-Visual (n = 12), and EOAD-Typical (n = 21) groups did not differ in age at onset, age at first evaluation, or age at death, or sex distribution (**Table 4**). PCA patients had more years of education than either EOAD-Typical or EOAD-Visual patients (p<0.001). APOE genotype distribution differed (p = 0.003) across groups, with PCA patients less likely than either EOAD group to have an ε4 allele, but no difference between EOAD-Visual and EOAD-Typical.

**Table 4:** Expanded participant demographics, clinical characteristics and neuropathology

	**AD - high visuospatial** **(EOAD-typical) (N = 21)**	**AD - low visuospatial** **(EOAD-visual) (N = 12)**	**PCA (N = 12)**	**P value**
Age at evaluation	64.24 ± 3.86	62.44 ± 5.13	63.12 ± 5.72	0.555
Age at onset	58.43 ± 3.16	56.17 ± 3.1	57.58 ± 4.87	0.166
Age at death	70.34 ± 5.15	67.54 ± 5.62	69.11 ± 6.41	0.389
Baseline – death interval	6.1 ± 2.32	5.11 ± 2.75	5.99 ± 3.23	0.581
Duration (onset to death)	11.91 ± 4.14	11.38 ± 3.67	11.52 ± 3.9	0.925
Female	10 (48 %)	5 (42 %)	2 (17 %)	0.255
Education	14.14 ± 2.59	13.83 ± 2.79	17.67 ± 1.37	< 0.001^b,c^
APOE ε4 alleles: 0 /1 / 2	6 / 13 / 2 (29 % / 62 % / 10 %)	5 / 6 / 1 (42 % / 50 % / 8 %)	11 / 1 / 0 (92 % / 8 % / 0 %)	0.003^b,c^
Predominant cognitive deficit at presentation: memory / language / executive / visuospatial	20 / 0 / 1 / 0 (95 % / 0 % / 5 % / 0 %)	9 / 2 / 0 / 1 (75 % / 17 % / 0 % / 8 %)	0 / 0/ 0 / 12 (0 % / 0 % / 0 % / 100 %)	< 0.001^b,c^
Clinical diagnosis: AD / LBD / other	20 / 1 / 0 (95 % / 5 % / 0 %)	9 / 3 / 0 (75 % / 25 % / 0 %)	9 / 1 / 2 (75 % / 8 % / 17 %)	0.08
MMSE	20.95 ± 5.29	16.82 ± 5.4	24.91 ± 4.25	0.003^b,c^
DRS	110 ± 15.96	95 ± 21.04	116.82 ± 12.35	0.021^a,c^
CDR sum of boxes	5.72 ± 2.24	7.88 ± 4.37	6 ± 5.28	0.462
Severe AD (Thal 4–5, Braak V–VI, CERAD mod.-frequent)	21 (100 %)	12 (100 %)	12 (100 %)	-
Lewy Body Disease: none / brainstem / limbic / neocortical	20 / 1 / 0 / 0 (95 % / 5 % / 0 % / 0 %)	10 / 2 / 0 / 0 (83 % / 17 % / 0 % / 0 %)	11 / 1 / 0 / 0 (92 % / 8 % / 0 % / 0 %)	0.798
TDP-43: none / amygdala / limbic / neocortical	13 / 5 / 3 / 0 (62 % / 24 % / 14 % / 0 %)	10 / 1 / 1 / 0 (83 % / 8 % / 8 % / 0 %)	12 / 0 / 0 / 0 (100 % / 0 % / 0 % / 0 %)	0.16
Cerebral amyloid angiopathy: none / mild / moderate / severe	2 / 4 / 11 / 4 (10 % / 19 % / 52 % / 19 %)	0 / 4 / 5 / 3 (0 % / 33 % / 42 % / 25 %)	2 / 1 / 7 / 2 (17 % / 8 % / 58 % / 8 %)	0.70
Infarct/microinfarct	2 (10 %)	2 (17 %)	2 (17 %)	0.73
Atherosclerosis of the circle of Willis: none / mild / moderate / severe	9 / 7 / 3 / 2 (43 % / 33 % / 14 % / 10 %)	6 / 3 / 3 / 0 (50 % / 25 % / 25 % / 0 %)	5 / 6 / 0 / 1 (42 % / 50 % / 0 % / 8 %)	0.55
Arteriolosclerosis: none / mild / moderate / severe	13 / 5 / 3 / 0 (62 % / 24 % / 14 % / 0 %)	9 / 1 / 1 / 1 (75 % / 8 % / 8 % / 8 %)	8 / 2 / 2 / 0 (67 % / 17 % / 17 % / 0 %)	0.78

a. Significant Post-hoc difference between EOAD-Typical vs EOAD-Visual. b. Significant Post-hoc difference between EOAD-Typical vs PCA. c. Significant Post-hoc difference between EOAD-Visual vs PCA. Demographic characteristics (mean and standard deviation), apolipoprotein E (APOE) ε4 status (frequency of ε4 alleles), duration of disease (years) and interval between baseline assessment and death (years) (mean and standard deviation), first type of symptom and predominant cognitive deficit at presentation (frequency of each), clinical diagnosis (frequency of Alzheimer’s disease (AD), Lewy body dementia (LBD), or other) and scores (mean and standard deviation) on the Mini-Mental State Exam (MMSE), Dementia Rating Scale (DRS) and Clinical Dementia Rating (CDR) sum of boxes at baseline shown separately for participants with early onset (typical) AD with relatively good visuospatial function (AD - High Visuospatial) or significantly impaired visuospatial function (AD - Low Visuospatial) or posterior cortical atrophy (PCA). Also shown are the number (and percentage) of individuals in each group with severe AD pathology, infarct / microinfarcts, or various levels of Lewy body disease, TDP-43, cerebral amyloid angiopathy, atherosclerosis, or arteriosclerosis pathology at autopsy. P-values for group comparisons with ANOVA are shown and pair-wise group differences are indicated with superscripts. For categorical variables, Fisher exact test is used. EOAD = Early Onset Alzheimer’s Disease.

The EOAD-Visual and PCA patients had higher OC NFT density and higher OC/MF NFT ratios than EOAD-Typical patients (all p < 0.05), while only EOAD-Visual had higher OC/HC NFT ratios (p = 0.008) (**Figure 3B, 3C**). The three groups did not differ significantly in MF or HC NFT density or in the MF/HC NFT ratio. The degree of concomitant Lewy body disease, TDP-43 pathology, and all vascular pathologies did not differ across groups (**Table 4**).

The three groups did not differ in type of first symptom at onset (i.e., cognition, behavior, or motor), but did differ in the predominant cognitive domain first affected (p < 0.001). 100 % of PCA patients, 8 % of EOAD-Visual, and 0 % of EOAD-Typical patients presented with visuospatial impairment. 95 % of EOAD-Typical patients and 75 % of EOAD-Visual patients presented with memory impairment; 17 % of EOAD-Visual patients presented with language impairment; and 5 % of EOAD-Typical patients presented with executive dysfunction. Interestingly, despite similar Visuospatial domain scores, PCA patients had significantly better Executive domain scores than either EOAD-Visual (β = 1.67 ± 0.40, p < 0.001) or EOAD-Typical (β = 1.41 ± 0.36, p < 0.001) patients. They also had better Memory domain scores than EOAD-Typical (β = 1.35 ± 0.20, p < 0.001) and EOAD-Visual (β = 0.75 ± 0.22, p = 0.002) patients. EOAD-Visual patients also had significantly better Memory domain scores than EOAD-Typical patients (β = 0.60 ± 0.19, p = 0.003) but similar executive domain scores (p = 0.44).

## Discussion

 We examined absolute and relative density of NFTs in occipital primary visual cortex versus hippocampus in patients with sporadic EOAD who initially presented with PCA or non-PCA syndromes. Consistent with previous results^20,21^, patients with PCA had lower NFT density than non-PCA patients in hippocampus and trended towards higher NFT density in occipital cortex. When NFT neuroanatomic distribution was quantified in ratios, patients with PCA had higher OC/HC and OC/MF ratios than non-PCA patients. The groups did not differ in MF/HC NFT density, highlighting disproportionate OC involvement in the PCA group, consistent with previous findings^22^. Severity of visuospatial deficits in the overall sample correlated with OC/HC and OC/MF NFT density ratios, including in the non-PCA group examined alone. These results suggest that occipital NFT pathology may be a major driver of visuospatial dysfunction across all patients with EOAD. 

 Recent clinicopathologic studies broadly support that PCA reflects posterior-predominant AD neurodegeneration but have not fully resolved how occipital pathology, medial temporal involvement, and cognitive phenotype are related. Our findings address this gap by showing that higher occipital-to-hippocampal NFT burden is associated with worse visuospatial performance across both PCA and non-PCA EOAD. Our findings bridge autopsy studies showing greater posterior cortical NFT burden in PCA versus typical AD with imaging and neuropsychological studies indicating that visuospatial impairment varies continuously across PCA and typical AD. Our work is also in line with a recent digital pathology study by Abdi et al.^24^ that found increased parietal tau and Aβ burden in PCA-AD compared with amnestic AD though they did not assess medial temporal AD pathology. While Abdi et al. included a high, differentially distributed burden of co-occurring α-synuclein pathology, our study excluded cases with neocortical or limbic α-synuclein pathology to better isolate the relationship between AD NFT topography and cognitive phenotype in EOAD. 

 Diagnostic criteria for PCA^6^ require insidious onset and gradual progression of prominent visuospatial dysfunction with relative sparing of other cognitive domain functions. The consensus framework also recognizes PCA-plus, in which patients meet the core PCA syndrome criteria but additionally fulfill criteria for another neurodegenerative syndrome. Thus, PCA-plus acknowledges phenotypic overlap within the PCA spectrum but does not provide a concrete definition for cases like our EOAD-Visual group. Like patients with PCA, our EOAD-Visual patients had a high degree of posterior pathology, an NFT distribution very similar to PCA, and a clear relationship between occipital NFT density and visuospatial dysfunction. These findings support the idea that PCA is one end of a continuum of cognitive-neuroanatomical changes relating visuospatial deficits to distribution of NFT pathology in EOAD^29,41,42^. They highlight that current diagnostic criteria may exclude ADNC patients with early, severe visuospatial deficits and disproportionate OC NFT burden, even when their clinicopathologic profile overlaps substantially with PCA. 

 Several factors may have contributed to EOAD-Visual patients not receiving a diagnosis of PCA despite prominent visuospatial dysfunction. First, only one EOAD-Visual patient reported visual impairment as the predominant symptom at onset, and this patient did not meet other criteria for PCA. It is possible that certain visual symptoms at onset (e.g., difficulty navigating or misidentifying objects) may be misreported as "memory problems" by patients or informants. Second, patients may not have had access to ophthalmologists who often provide first diagnosis of PCA when visual symptoms are predominant^43^ and did not present for cognitive evaluation until additional symptoms (e.g., memory decline) were apparent. Third, EOAD-Visual patients had significantly impaired Executive cognitive domain scores that did not differ from those of EOAD-typical patients. In contrast, the Memory and Executive domain scores of PCA patients were above the average of age-matched robust normal controls, accentuating their disproportionate visual impairment. It should be noted that no PCA patients had concomitant TDP-43 pathology, while subcortical TDP-43 pathology was present in 30 % of the non-PCA early onset patients (38 % of the EOAD-Visual patients). TDP-43 pathology's effect on cognition, especially before its neocortical stage, is largely confined to memory, so it is possible that additional memory impairment due to TDP-43 pathology may have masked the visuospatial impairments in the EOAD-Visual cohort. If this is the case, these individuals may have satisfied level 2 criteria for PCA-plus^6^. 

 The unknown factors that predispose one to selective occipital cortex vulnerability may not be unique to those with the PCA syndrome but may also occur in typical memory-predominant AD with severe visuospatial dysfunction. These could include genetic risks for PCA that may be shared by some individuals with non-PCA EOAD. A genome wide association study found that variation in or near *APOE/TOMM40* increased PCA risk, but with smaller risk than for typical AD^13^. There was evidence for risk in or near *CR1*, *ABCA7* and *BIN1*, while odds ratios at variants near *INPP5D* and *NME8* did not overlap between PCA and typical AD. Other studies have identified genetic loci associated with isolated substantial visuospatial impairment in genome-wide single-nucleotide polymorphism (SNP) data from patients with late onset AD, including variants in or near *NIT2*, *SPATS1*, *CSMD1*, and ^44^*SLC14A2*. Similarly, a selective increase in neuronal genomic mosaicisms in occipital cortex^45^ could be shared by PCA and Visual-EOAD. Future research is needed to determine factors that could cause selective vulnerability of occipital cortex across the AD spectrum. 

 Our finding that some EOAD patients have a distribution of NFT more similar to PCA than typical amnestic AD has several implications for clinical trials, including recent trials that led to approval of anti-amyloid therapies^46,47^. First, eligibility criteria for these trials required memory impairment, excluding many patients with atypical presentations of sporadic EOAD. Thus, the efficacy of these anti-amyloid agents in patients with PCA remains unknown. Second, the primary and secondary outcome measures used in these trials (e.g., CDR sum of boxes, ADAS-cog, ADCOMS) are heavily weighted towards memory function and may not be sensitive to effects in patients with PCA, even though their underlying pathology is AD. Even when individuals with PCA are excluded, patients like those in our EOAD-Visual group have memory impairment that may qualify them for these trials, but their greater occipital to hippocampal NFT distribution may reduce the ability of standard memory-weighted outcome measures to track progression and drug response in these patients. Tracking changes in tau-PET measures with regions of interest designed for typical amnestic AD raises similar concerns. The response of EOAD-Visual patients to disease modifying therapy may be more similar to PCA patients than those with typical EOAD. Clinical trials that include PCA or EOAD-Visual patients should utilize outcome measures that assess visuospatial abilities in addition to memory function. 

 The strengths of this study include well characterized cohorts of PCA and non-PCA EOAD patients with extensive baseline neuropsychological testing that allowed creation of robust domain scores. They also had detailed neuropathological assessment that allowed verification of ADNC, identification of other forms of potentially confounding pathology (e.g., LBD, TDP-43), and determination of relative density of NFT pathology in the hippocampus and various cortical regions. A limitation of the study is the relatively small sample size, which is not unexpected given that PCA is a relatively rare diagnosis. Despite the small sample size this is one of the largest samples of PCA cases evaluated histologically for distribution of tau NFTs. A second limitation is the delay between the baseline clinical assessment and autopsy. The pattern of cognitive deficits and their relationship to NFT pathology may change over time in PCA and non-PCA patients weakening the association. Evidence from longitudinal studies comparing cognitive changes and hippocampal/cortical atrophy on MRI in PCA and typical AD cases suggests that both groups experience widespread decline that could increase similarities between groups, although distinct (but possibly attenuated) patterns are maintained over time^48^. A third limitation is that the patients with PCA had a higher level of education than the non-PCA patients. This complicates interpretation of group differences since education may provide a greater protective effect (e.g., cognitive reserve) in memory and executive domains that are relatively preserved in PCA than in the more vulnerable visuospatial domain. A final limitation is that, in this legacy cohort, relatively newer pathologic entities such as argyrophilic grain disease (AGD) and age-related tau astrogliopathy (ARTAG) were not systematically staged, though both pathologies are rare in the younger age range of our study population. 

In conclusion, our results demonstrate a strong relationship between tau NFT pathology in the occipital cortex and visuospatial impairment in sporadic EOAD. Patients with severe visuospatial deficits had a high OC/HC distribution of NFT pathology regardless of whether or not they satisfied clinical criteria for PCA. Thus, cognitive testing may identify patients with substantial occipital NFT pathology that might influence cognitive and imaging outcome measures in clinical trials of AD modifying therapies.

## Conflict of interest statement

Annie Hiniker has served on an advisory board for Siemens Healthineers and consulted for PrecisionMed. Douglas Galasko has consulted for Eisai, Actinogen, Cognition Therapeutics, and served on a DSMB for Artery Therapeutics. David Salmon has consulted for Aptinyx and Biogen. Denis Smirnov, Melanie Estrella, Sophie Dickinson, Vanessa Goodwill, Alison Headley and Donald Pizzo declare no competing interests.

## Supplementary material

Supplemental Table 1 (PDF file, 331 KB).

## Supplementary Material

Supplemental Table 1 (PDF file, 331 KB)
