# Supplementary material for: Neurofibrillary tangle distribution in posterior cortical atrophy and typical early-onset Alzheimer's Disease
Source: Free Neuropathol. 2026 Jul 9;7:18. doi: 10.17879/freeneuropathology-2026-9686 (PMC13366614; doi:10.17879/freeneuropathology-2026-9686)
Supplement: Supplementary file 1 [file freeneuropathol-07-18-9686-s1.pdf]

DOI: https://doi.org/10.17879/freeneuropathology-2026-9686

Supplementary Table 01

Domain	Predictor	β ± SE (All)	p (All)	β ± SE (EOAD)	p (EOAD)
Memory	**HC Tangles**	**−0.018 ± 0.006**	**0.010**	**−0.014 ± 0.006**	**0.034**
	MF Tangles	−0.006 ± 0.010	0.531	−0.007 ± 0.009	0.466
	OC Tangles	0.010 ± 0.010	0.340	0.012 ± 0.012	0.313
	MF/HC Ratio	0.505 ± 0.276	0.075	0.278 ± 0.297	0.358
	**OC/HC Ratio**	**0.573 ± 0.241**	**0.023**	**0.630 ± 0.232**	**0.012**
	OC/MF Ratio	0.317 ± 0.207	0.134	0.399 ± 0.235	0.102
Executive	HC Tangles	0.015 ± 0.012	0.201	**0.018 ± 0.009**	**0.049**
	**MF Tangles**	**−0.038 ± 0.015**	**0.019**	**−0.033 ± 0.012**	**0.010**
	OC Tangles	−0.003 ± 0.018	0.867	−0.014 ± 0.016	0.419
	**MF/HC Ratio**	**−1.163 ± 0.438**	**0.012**	**−1.186 ± 0.356**	**0.003**
	OC/HC Ratio	−0.522 ± 0.451	0.255	−0.629 ± 0.353	0.087
	OC/MF Ratio	0.326 ± 0.374	0.390	0.214 ± 0.349	0.546
Visuospatial	HC Tangles	0.010 ± 0.013	0.453	0.007 ± 0.013	0.594
	MF Tangles	−0.006 ± 0.018	0.759	−0.003 ± 0.019	0.891
	**OC Tangles**	**−0.050 ± 0.017**	**0.007**	**−0.060 ± 0.020**	**0.005**
	MF/HC Ratio	−0.329 ± 0.520	0.531	−0.218 ± 0.594	0.717
	**OC/HC Ratio**	**−1.105 ± 0.468**	**0.024**	**−1.288 ± 0.454**	**0.009**
	**OC/MF Ratio**	**−0.927 ± 0.384**	**0.021**	**−1.218 ± 0.425**	**0.008**

Table S1: Association of regional tangle burden and cognitive performance adjusted for APOE genotype and TDP-43 proteinopathy

Standardized regression coefficients (β) and p-values from linear regression models examining the association between neurofibrillary tangle (NFT) density (tangles per high-power field, /HPF) in the hippocampus (HC), middle frontal gyrus (MF), and occipital cortex (OC), and NFT density ratios (MF/HC, OC/HC, OC/MF), with composite scores for Memory, Executive, and Visuospatial cognitive domains. Cognitive composites were derived from principal component analysis of 16 neuropsychological tests administered at baseline (**Table 2**). All models were adjusted for age at assessment, sex, education, the interval between the baseline assessment and death (years), as well as the APOE ε4 carrier status (≥ 1 ε4 allele) and TDP-43 status (any LATE stage vs none). Compare to Table 3 (unadjusted for APOE or TDP-43). Results are shown separately for the full cohort (early onset AD and posterior cortical atrophy combined) and for participants with early onset (typical) AD only.

freeneuropathology.org

Copyright: © 2026 The author(s). This is an open access article distributed under the terms of the Creative Commons Attribution 4.0 International License (https://creativecommons.org/licenses/by/4.0/), which permits unrestricted use, distribution, and reproduction in any medium, provided the original author and source are credited, a link to the Creative Commons license is provided, and any changes are indicated. The Creative Commons Public Domain Dedication waiver (https://creativecommons.org/publicdomain/zero/1.0/) applies to the data made available in this article, unless otherwise stated.
